# Bilateral Globus Pallidus Interna Combined With Subthalamic Nucleus Variable Frequency Deep Brain Stimulation in the Treatment of Young-Onset Parkinson's Disease With Refractory Dyskinesia: A Case Report

**DOI:** 10.3389/fnins.2021.782046

**Published:** 2021-11-25

**Authors:** Bowen Chang, Jiaming Mei, Chi Xiong, Peng Chen, Manli Jiang, Chaoshi Niu

**Affiliations:** ^1^Division of Life Sciences and Medicine, Department of Neurosurgery, The First Affiliated Hospital of USTC, University of Science and Technology of China, Hefei, China; ^2^Anhui Provincial Stereotactic Neurosurgical Institute, Hefei, China; ^3^Anhui Key Laboratory of Brain Function and Brain Disease, First Affiliated Hospital of University of Science and Technology of China, Hefei, China

**Keywords:** Parkinson's disease, dyskinesia, deep brain stimulation (DBS), treatment, subthalamic nucleus

## Abstract

**Background:** Main motor characteristics in Parkinson's disease (PD) include bradykinesia, rigidity, and tremors. With the development of neuromodulation techniques, it has become possible to use deep brain stimulation (DBS) to control the symptoms of PD. However, since the subthalamic nucleus(STN) and globus pallidus interna (GPi) DBS have their own advantages and disadvantages, it is difficult to control symptoms of the patients. It is essential to find new stimulation methods.

**Case Presentation:** A 33-year-old male PD patient with onset at the age of 12 years. The onset of the disease is presented with bradykinesia and progressively developed severe choreic dyskinesia with the use of medications. We then performed a thorough evaluation of the patient and decided to perform bilateral globus pallidus interna combined with subthalamic nucleus variable frequency DBS (bSGC-DBS) implantation, and after 2 years of follow-up the patient's bradykinesia and dyskinesia symptoms and quality of life improved significantly.

**Conclusions:** This is the first case of bSCG-DBS in a PD patient with refractory dyskinesia, and the first report of encouraging results from this clinical condition. This important finding explores multi-electrode and multi-target stimulation for the treatment of dystonia disorders.

## Background

Main motor characteristics in Parkinson's disease (PD) include bradykinesia, rigidity, and tremors. These characteristics, with the exception of variable tremors, can be significantly improved through levodopa treatment. This is especially beneficial early in the disease, wherein improvements can be retained through intermittent dosing in waking hours. In contrast, dopamine replacement therapy in PD has been associated with several medication-induced complications, of which dyskinesia is considered to be the most baneful influence. As such, a decreased levodopa dose with shortened dosage time intervals has been the most commonly used method for dyskinesia treatment, although some cases can still be difficult to manage (Benabid et al., 2000). Therefore, the development of new therapeutic interventions to reduce the impact of dyskinesia in PD is an important need.

## Case Presentation

**Figure 1:**
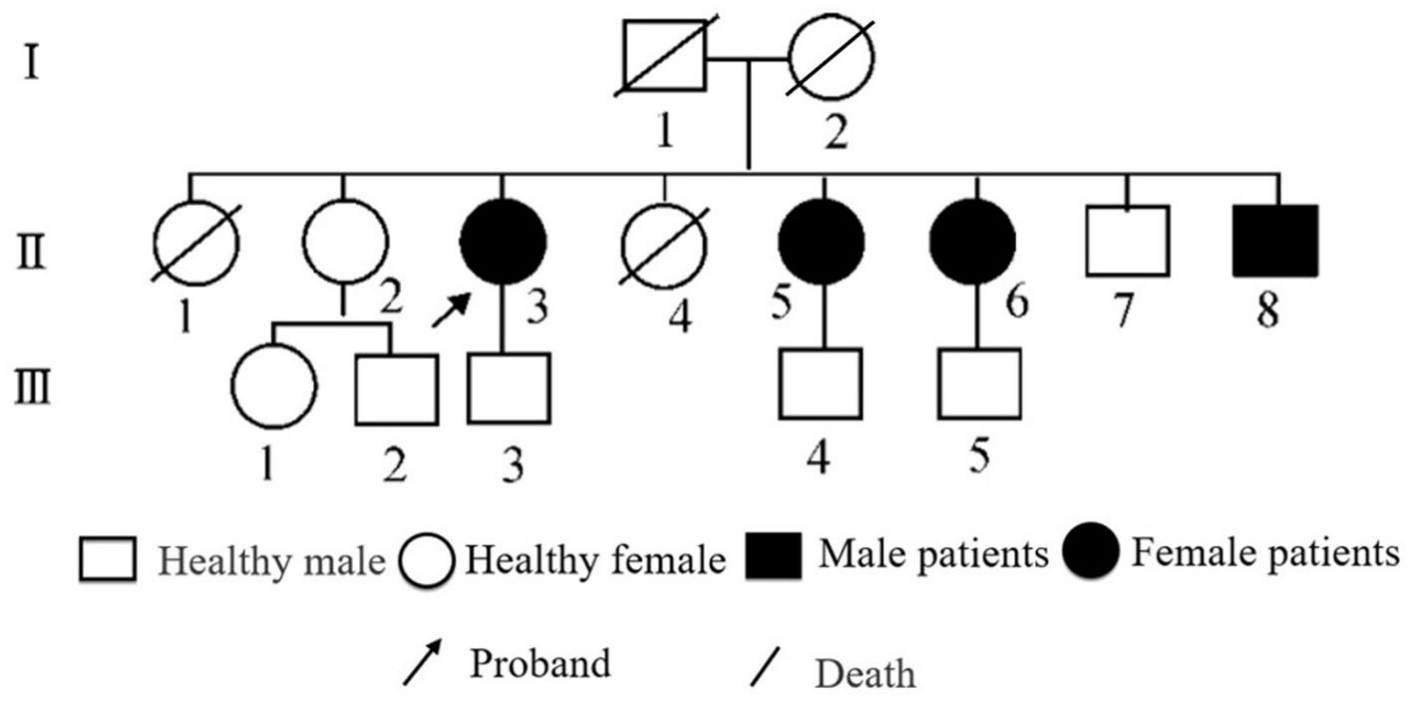
Genetic family diagram of the patient.

The patient was a 33-year-old man who presented with unsteady gait, limb tremors, and bradykinesia at the age of 12 years. Initially, the patient was treated with oral levodopa and benserazide, amantadine, and benzhexol, resulting in an improvement in his symptoms. However, these symptoms gradually worsened, and the dosage was gradually increased. Despite this, the symptoms could not be controlled, and the patient gradually developed severe chorea-like dyskinesia of the extremities. Furthermore, 5 years after taking the drug, during the drug-off period, the patient had significant bradykinesia and was unable to take care of himself. On assessment of the patient's family history, it was found that the patient's two sisters had similar symptoms (Figure 1). As a result, the patient and his two sisters underwent genetic testing for a single-gene genetic disorder. We found heterozygous mutations in the PRKN gene in the patient and his two sisters, leading to a diagnosis of “familial hereditary young-onset PD.” (Supplementary Material).

A series of examinations was performed on the patient before surgery. Cranial magnetic resonance imaging (MRI) revealed no significant abnormalities other than mild brain atrophy. Cranial PET-CT showed decreased 18F-Dopa concentration in both the posterior putamen and decreased FDG metabolism in the left frontal temporal caudate nucleus head, left inferior parietal gyrus, and right posterior middle lobe gyrus. Electroencephalography showed no abnormalities.

The patients were assessed with a detailed scale to assess the severity of their symptoms, psychological status, and quality of life. The UPDRS-III, UPDRS-IV scale is used to assess the severity of the patient's symptoms. The NMSS (Parkinson's Non-motor Symptom Scale) is an assessment of Parkinson's non-motor symptoms. The PDQ-39 scale was used to evaluate the quality of life and psychological state of the patients. We found that the patient had severe bradykinesia and dyskinesia.

Previous randomized controlled trials (RCTs) comparing STN-DBS with GPi-DBS have demonstrated the beneficial role of STN-DBS in medication reduction and the role of GPi-DBS in the reduction of dyskinesia severity. However, high frequency stimulation of the STN in PD patients can induce intense dyskinesias that are similar to those induced by levodopa. The patient was unable to lower his medication because of intractable bradykinesia and had severe choreic dyskinesia. After discussion, our team concluded that neither the single use of bilateral STN-DBS nor the single use of GPi-DBS could improve all symptoms of the patient.

We then decided to perform bSGC-DBS implantation. The DBS electrode (model PINS G102R-new; PINS Medical Co., Ltd, Beijing, China) was implanted into the bilateral STN(L301) and GPi(L302).

**Figure 2:**
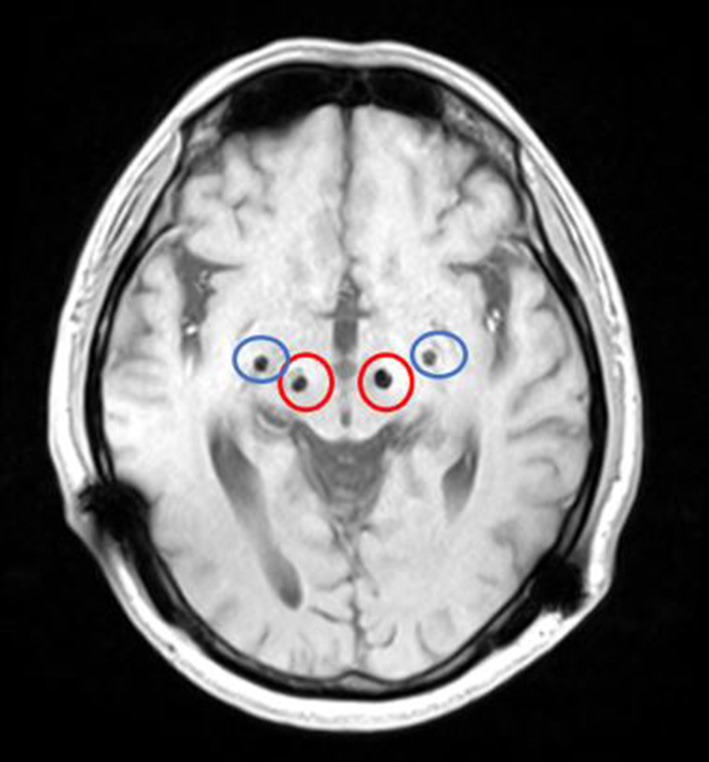
MRI (magnetic resonance imaging) after bSCG-DBS implantation (the red circle is STN, and the blue circle is GPi).

**Figure 3:**
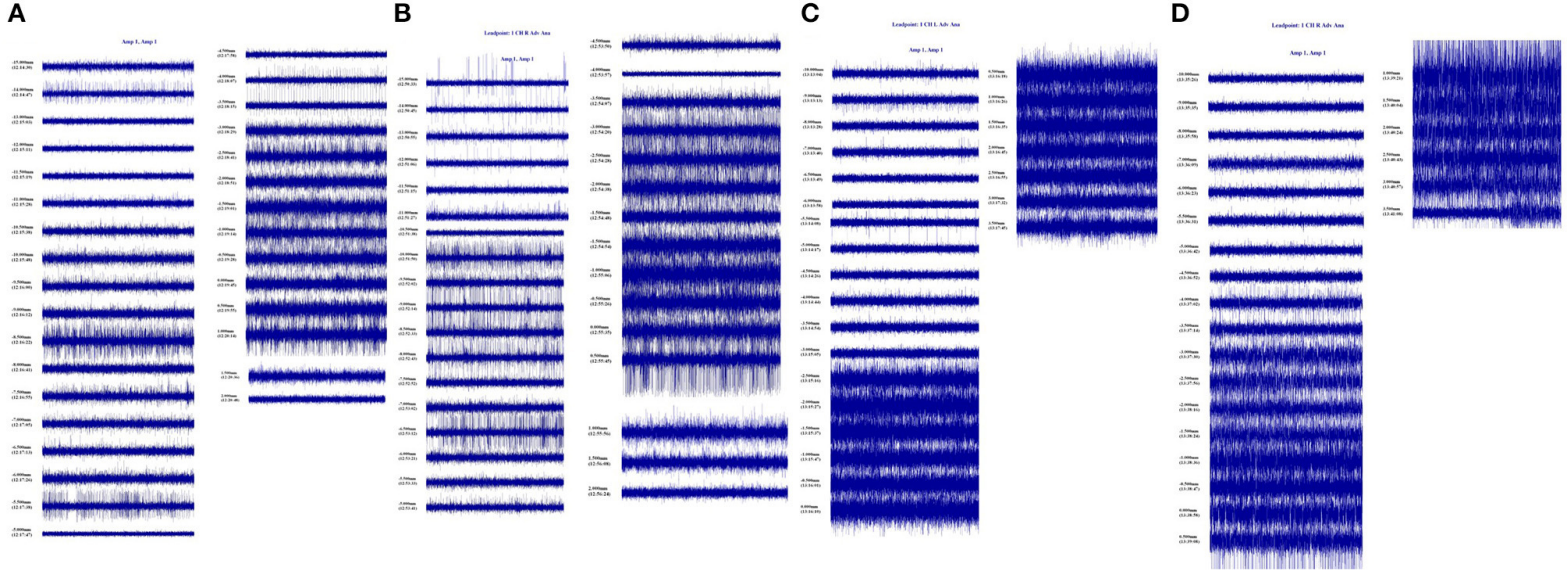
Intraoperative microelectrode monitoring was recorded. **(A)** GPi on the left: during the monitoring process, GPe electrical activity was recorded from 11.0 mm above the target to 5.5 mm above the target, GPi discharge was recorded from 4.0 mm above the target to 1.5 mm below the target, and the electrode was placed at 1.0 mm below the target. **(B)** GPi on the right side: during the monitoring process, GPe electrical activity was recorded from 11.0 to 4.5 mm on the target, and GPi discharge was recorded from 3.5 to 1.5 mm below the target; The electrode was placed 1.0 mm below the target. **(C)** left STN: electrical activity of STN is recorded from 2.5 mm above the target to 3.5 mm below the target during monitoring: electrode is placed at 3.0 mm below the target. **(D)** Right STN: electrical activity of STN was recorded from 4.0 mm above target to 3.0 mm below target during monitoring; The electrode was placed 2.5 mm below the target.

We conducted pre-operative head placement of the patient with a Leksell-G orientation instrument, followed by an MRI scan. According to the scanning results, the frame coordinates of GPi were determined as (left: *X* = 126.5, *Y* = 107, *z* = 116. 5; right: *x* = 78, *y* = 107, *z* = 116.5) and the frame coordinates of STN (left: *x* = 114.5, *y* = 104, *z* = 118.5; right: *x* = 88.5, *y* = 104, *z* = 118.5). The implantation point of the GPi was posterior, 28 mm from the implantation point of the STN (Figure 2). Microelectrode monitoring was performed to determine the location of the target (Figure 3). After electrode implantation, temporary intraoperative test stimulation was applied to the patient, and the stimulation parameters were adjusted to 3.5 V voltage, 90 us pulse width, and 150 Hz frequency, when the patient reached a comfortable state. During the temporary stimulation, there was no visual tract or internal capsule stimulation and no nausea or adverse speech reaction.

**Figure 4:**
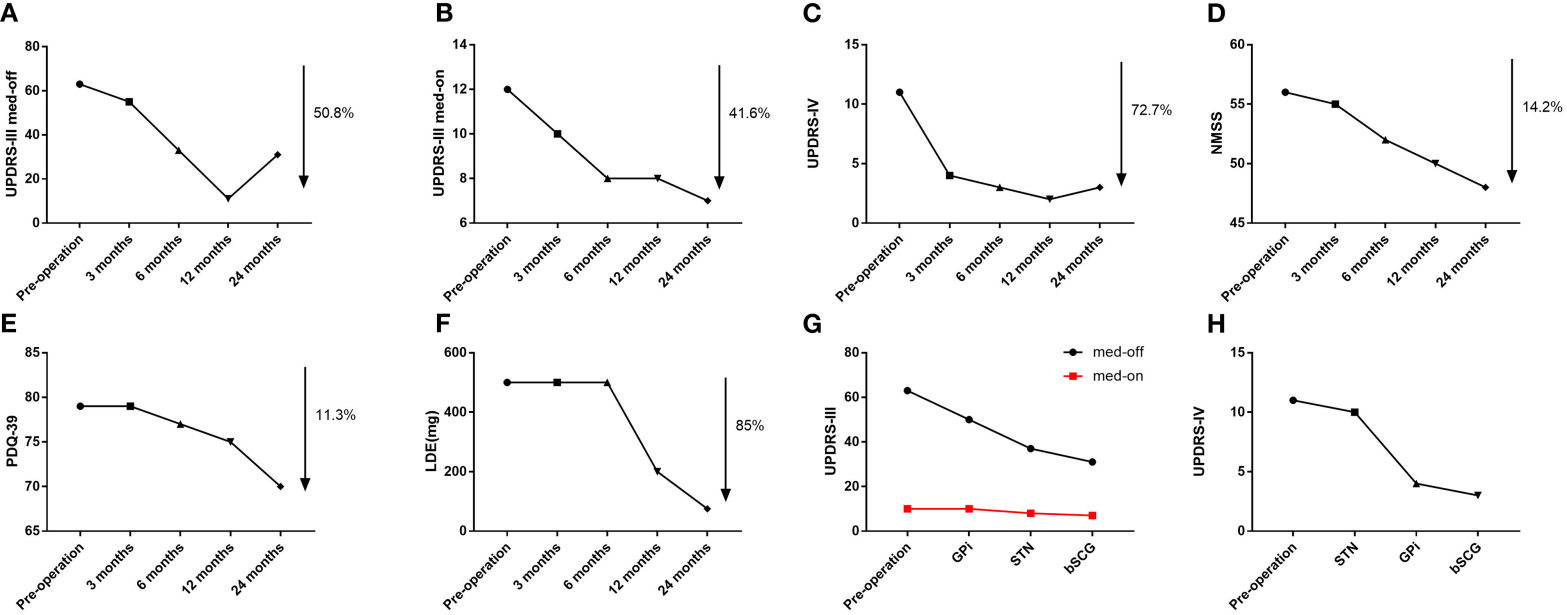
**(A)** Trends in UPDRS-III off-period scores. **(B)** Trends in UPDRS-III on-period scores. **(C)** Trends in UPDRS-IV scores. **(D)** Trends in NMSS scores. **(E)** Trend in PDQ-39 scores. **(F)** Trends in Levodopa Equivalents (LDE). **(G)** Comparison of UPDRS-III score in 4 conditions (pre-surgery, GPi stimulation, STN stimulation, and combined GPi and STN stimulation). **(H)** Comparison of UPDRS-IV score in 4 conditions (pre-surgery, GPi stimulation, STN stimulation, and combined GPi and STN stimulation).

In the 2nd week after surgery, we performed the first parameter adjustment for the patient's DBS. First, we activated the bilateral electrodes of the GPi and started with the following parameters: 90 μs for pulse width, 125 Hz for frequency, and 3.0 V for voltage. When we tried to adjust the parameters upwards, the patient's bilateral lower extremity dyskinesia became apparent. When we reduced the GPi pulse width to 70 μs, the patient's lower extremity dyskinesia disappeared, but the bradykinesia was still present. Electrodes in the STN were not activated when the parameters were first adjusted. In the 3rd month after surgery, we performed a second parameter adjustment. This time we made a slight adjustment in the GPi parameters, and the patient showed good control of dyskinesia without fluctuating symptoms. However, since the patient's bradykinesia did not improve with GPi-DBS alone, we subsequently activated the electrodes in the bilateral STN, with the following adjusted parameters: 70 μs for pulse width, 130 Hz for frequency, and 2.0 V for voltage. As a result, the patient's limb dyskinesia improved significantly, and bradykinesia also improved. In the 6th month after surgery, we performed the third parameter adjustment. At this time, the overall condition of the patient improved, as compared to the previous one. Because previous studies have shown that STN-DBS variable frequency stimulation (VFS) can increase gait speed and reduce the number of freezing episodes (Follett et al., 2010). For this adjustment, we adjusted the stimulation mode of the STN to VFS with parameters of 90, 105, 125, and 105 Hz, alternating every 0.1 s. In the 12th month after surgery, the patient showed further improvement in walking and limb dyskinesia, when compared to that in the previous period, and dyskinesia had been largely controlled. We performed the fourth parameter adjustment by adjusting the VFS mode parameters of STN to the following: 90 Hz-2 s; 130 Hz-7 s; 160 Hz-7 s; and 170 Hz-2 s. In the following 12 months, no parameter adjustment was performed, since the patient's symptoms were stable. Specific adjustment parameters are presented in Supplementary Material. At 24 months after surgery, the patient's dyskinesia symptoms had largely disappeared, and the bradykinesia was significantly improved. The dosage has also dropped dramatically (Figures 4A–F). Furthermore, the patient noted that he was able to take care of himself and was very satisfied with the overall outcome. We attempted to switch STN and GPi on and off alternately, subsequently evaluating patients' UPDRS-III and UPDRS-IV scores (Figures 4G,H). In the UPDRS-IV scale, the score of dyskinesia part was 6 before surgery, 2 in 3 months after surgery, 1 in 6 months after surgery, 1 in 12 months after surgery, and 2 in 24 months after surgery.

## Discussion and Conclusions

With the development of neuromodulation techniques over the past 30 years, deep brain stimulation (DBS) has been found to improve levodopa-responsive symptoms, dyskinesia, and tremors. Subthalamic nucleus DBS (STN-DBS) and globus pallidus interna DBS (GPi-DBS) have now been assessed by new studies to be valid in dyskinesia treatment, showing clinical significance in practice (Fox et al., 2018). However, regarding treatment outcomes, numerous studies have verified the absence of remarkable discrepancies between these two targets, although controversies still exist about their respective treatment outcomes (Jia et al., 2018). Moreover, previous RCTs comparing STN-DBS with GPi-DBS have demonstrated the conducive role of STN-DBS in medication reduction and GPi-DBS in the reduction of dyskinesia severity (Sharma et al., 2010; Mansouri et al., 2018). However, High-frequency stimulation of the STN in PD patients can induce intense dyskinesias that are similar to those induced by levodopa (Vincent et al., 2016). Hence, the goals of DBS could be of great importance for target selection.

Based on the respective advantages of STN-DBS and GPi-DBS, this study developed bilateral STN-DBS in combination with GPi-DBS (bSCG-DBS) for the treatment of PD with severe dyskinesia. Furthermore, it was found that this new stimulation mode had better efficacy than STN-DBS or GPI-DBS alone. Herein, we report the first case of bSCG-DBS treatment in a patient with familial hereditary young-onset Parkinson's disease with refractory dyskinesia.

In this case report, STN stimulation was more effective for reducing bradykinesia, while GPi stimulation was more effective for reducing dyskinesia. Although in RCTs that STN DBS and GPI DBS are equally effective for the treatment of parkinsonism, in this case report we found that STN DBS was more effective for the reduction of bradykinesia, while GPI DBS was more effective for the reduction of dyskinesia (Wong et al., 2019). In conclusion, STN stimulation appears to be insufficient to control refractory choreiform dyskinesia; therefore, the combination of GPi and STN stimulation provided some moderate advantage over STN/GPi stimulation alone.

To our knowledge, this is a case of bSCG-DBS in a PD patient with refractory dyskinesia, and the report of satisfactory results from this clinical condition. However, further studies are needed to confirm this important finding, which explores multi-electrode and multi-target stimulation for the treatment of PD.

## Data Availability Statement

The original contributions presented in the study are included in the article/Supplementary Material, further inquiries can be directed to the corresponding author.

## Ethics Statement

The studies involving human participants were reviewed and approved by the First Affiliated Hospital of USTC, Division of Life Sciences and Medicine. The patients/participants provided their written informed consent to participate in this study. Written informed consent was obtained from the individual(s) for the publication of any potentially identifiable images or data included in this article.

## Author Contributions

BC and JM jointly completed the experiment and the writing. CX, PC, and MJ are responsible for post-operative parameter regulation. CN took overall control of the whole study. All authors contributed to the article and approved the submitted version.

## Funding

This work was supported by the Special Fund Project for Guiding Local Science and Technology Development by the Central Government (No: 2019b07030001).

## Conflict of Interest

The authors declare that the research was conducted in the absence of any commercial or financial relationships that could be construed as a potential conflict of interest.

## Publisher's Note

All claims expressed in this article are solely those of the authors and do not necessarily represent those of their affiliated organizations, or those of the publisher, the editors and the reviewers. Any product that may be evaluated in this article, or claim that may be made by its manufacturer, is not guaranteed or endorsed by the publisher.
