# Supplementary material for: Bilateral Globus Pallidus Interna Combined With Subthalamic Nucleus Variable Frequency Deep Brain Stimulation in the Treatment of Young-Onset Parkinson's Disease With Refractory Dyskinesia: A Case Report
Source: Front Neurosci. 2021 Nov 25;15:782046. doi: 10.3389/fnins.2021.782046 (PMC8656942; doi:10.3389/fnins.2021.782046)
Supplement: Supplementary file 1 [file Table_1.DOCX]

**Specific adjustment parameters**

**1. The first program control （2 weeks after operation）**

**GPi**

|  |  | Pulse width (µs) | Frequency (Hz) | Voltage (V) |  |
| --- | --- | --- | --- | --- | --- |
| R | 3+2-  1+3- | 70us  90us | 125Hz | 3.0V  3.0V | The dyskinesia of lower limbs disappeared after the adjustment of the parameters, but the effect also gradually disappeared |
| L | 7+6-  5+7- | 70us  90us |  | 3.0V  3.0V |  |

**2. 3 months after operation**

**GPi**

|  |  | Pulse width (µs) | Frequency (Hz) | Voltage (V) |  |
| --- | --- | --- | --- | --- | --- |
| R | C+2-  C+3- | 60us  80us | 120Hz | 2.7V  3.6V | The patient had good movement of both upper limbs, no symptom fluctuation, and the walking was not ideal. After this parameter continued to be increased, abnormal peak movement appeared. |
| L | 7+6-  6+7- | 70us  70us |  | 2.9V  3.6V |  |

**STN**

|  |  | Pulse width (µs) | Frequency (Hz) | Voltage (V) |  |
| --- | --- | --- | --- | --- | --- |
| R | c+1- | 60us | 130Hz | 1.8V | Dizziness, right hand numbness and discomfort |
| L | c+5- | 60us |  | 1.9V |  |

|  |  | Pulse width (µs) | Frequency (Hz) | Voltage (V) |  |
| --- | --- | --- | --- | --- | --- |
| R | c+3- | 60us | 130Hz | 1.8V | After 10min, the effect of bradykinesia and pulse width increased by 10us was not obvious |
| L | c+7- | 60us |  | 1.8V |  |

|  |  | Pulse width (µs) | Frequency (Hz) | Voltage (V) |  |
| --- | --- | --- | --- | --- | --- |
| R | c+2- | 70us | 130Hz | 2.0V | No discomfort, limb activity improved significantly, walking improved |
| L | c+6- | 70us |  | 1.8V |  |

**3. 6 months after operation**

**GPi**

|  |  | Pulse width (µs) | Frequency (Hz) | Voltage (V) |  |
| --- | --- | --- | --- | --- | --- |
| R | C+2-  C+3- | 60us  80us | 120Hz | 2.8V  3.0V | Patients reported further improvement in walking and limb mobility, and better speech |
| L | 7+6-  6+7- | 70us  70us |  | 2.9V  3.5V |  |

**STN**

|  |  | Pulse width (µs) | Frequency (Hz) | Voltage (V) |  |
| --- | --- | --- | --- | --- | --- |
| R | C+2-  C+3- | 60us  70us | VFS | 2.4V  2.4V | VFS：90Hz-0.1s  105Hz-0.1s  125HZ-0.1s  105Hz-0.1s |
| L | C+6-  C+7- | 60us  70us |  | 2.4V  2.4V |  |

**4. 12 months after operation**

**GPi**

|  |  | Pulse width (µs) | Frequency (Hz) | Voltage (V) |  |
| --- | --- | --- | --- | --- | --- |
| R | C+2-  C+3- | 60us  80us | 125Hz | 2.6V  2.8V | The patient's upper limbs moved freely, speech was the same as before, and walking was ok, but there was still a freeze |
| L | 7+6-  6+7- | 70us  80us |  | 2.9V  3.3V |  |

**STN**

|  |  | Pulse width (µs) | Frequency (Hz) | Voltage (V) |  |
| --- | --- | --- | --- | --- | --- |
| R | c+2-3- | 60us | VFS | 2.5V | VFS：90Hz-2s  130Hz-7s  160HZ-7s  120Hz-2s |
| L | c+7- | 80us |  | 2.8V |  |
